# Hog Cholera on Long Island—Treatment by Chlorate of Potash

**Published:** 1881-04

**Authors:** 


### VI.—HOG CHOLERA ON LONG ISLAND.—TREATMENT BY CHLORATE OF POTASH.

J. Lindsay, D.V.S., of Huntingdon, L. I., has sent to the JOURNAL some interesting notes which are of special importance with reference to the question of contagious diseases among hogs in this country. Dr. Lindsay writes us, that on December 17th last he was called to the stock farm of Messers. Stehlin & Co., at Lloyd's Neck, Long Island, and asked to examine a herd ot one hundred and twenty-seven hogs, suffering, as Mr. Stehlin said, from an unknown disease, which he supposed was pneumonia. The following symptoms were gathered at the time: diminished appetite, drooping head and listless appearance, staring coat, and arched back. The eyes and nose discharged at first a watery fluid that subsequently became thick, purulent and sticky. This discharge accumulated around the eye-lids and nostrils. Sometimes the discharge was streaked with blood. Their bodies were covered either with red or purple spots, and backs of ears were very red. There was also a dry, hacking cough. Constipation was followed by diarrhœa, and the discharges were very offensive. The animals withdrew from their companions, and becoming emaciated and weakened, died in pain, in from four to twenty hours. Some few lived for two or three days. At the post-mortem examination of one that had died twelve hours previously, the blood was found fluid and of a dark coffee-color, coagulating very slowly after exposure to the air. The lungs were infiltrated with dark material thought to be blood, and looking like pulmonary apoplexies.

The right lung showed this condition most markedly. The trachea contained bloody froth, and the pleuræ exhibited patches of ecchymosis about the size of five-cent pieces. The heart was soft, flabby, and contained a small amount of blackish blood in the right ventricle, its internal structure being normal. The diaphragm, liver, and spleen and kidneys were normal. The mucous coat of the stomach was covered with large patches of ecchymosis; the large and small intestines also showed ecchymotic spots on their internal surfaces and in their muscular substance. The fœces had a dry, offensive odor. The anus was prolapsed. Blood had flowed from the mouth and nose after death.

Some light is thrown upon the cause of the disease by the following facts: During the summer Mr. Stehlin had been keeping a choice lot of Red Jerseys, Chester, Whites, and Blooded Yorkshires, eighty-seven in number. These animals, which were kept in a large yard with connecting pens, were fed upon diet a of milk and corn meal. Their condition had been excellent all the season. On December 13th he bought at Bull's Head in New York City a lot of forty picked black Berkshire sows. They appeared to be in fine condition, and there was no suspicion that they were in any way tainted, until, on Dec. 25th, four were found dead. Subsequently he remembered that a few had had a dry cough, but at the time it was thought to be merely from an ordinary cold, for which sulphur was given. On the morning of the 27th he found eleven dead, and becoming alarmed, he sent for a veterinarian. The black pigs came from the West by car, and presumably brought the disease, while they themselves were passing through the incubatory stage.

The treatment seems to have been measurably successful, and therefore deserves notice. First the whole herd was divided up into sections of ten each. The diet was changed from boiled turnips, potatoes and beets, to mashed potatoes and linseed meal. This allowance was given them twice daily, and each pig received at the same time potassium chlorate gr. xxx., with brandy, two ounces. Other forms of treatment had been tried by a veterinary surgeon from New York, who was disposed to regard the disease as a simple pneumonia from a cold. He had ordered vinegar and opium, magnesia sulphate, and tannic acid, regulating the medicine so as to control the constipation or diarrhœa. The mortality, however, was four to eight per day. Under the potassium chlorate treatment, it dropped to two to four per day, and in six weeks ceased altogether. Dr. L. states that though he would not like to call his drug a specific, it is fair to assume that it is a valuable remedy.

